# Long Noncoding XLOC_006390 Regulates the Proliferation and Metastasis of Human Colorectal Cancer via miR-296/ONECUT2 Axis

**DOI:** 10.1155/2022/4897201

**Published:** 2022-07-15

**Authors:** Tianyi Ma, Tianyu Qiao, Rui Huang, Meng Wang, Hanqing Hu, Ziming Yuan, Yihao Zhu, Hongyu Wu, Xiaoming Zou

**Affiliations:** ^1^Department of Colorectal Surgery, The Second Affiliated Hospital of Harbin Medical University, Harbin 150001, China; ^2^Department of General Surgery, The Second Affiliated Hospital of Harbin Medical University, Harbin150001, China

## Abstract

Long noncoding RNA (LncRNA) XLOC_006390 has been shown to be dysregulated in cancer tissues and regulates cancer growth and development. Nonetheless, the molecular role of lncRNA-XLOC_006390 in colorectal cancer via modulation of miR-296/ONECUT2 axis is still unclear. Against this backdrop, the current study was designed to explore the role of lncRNA-XLOC_006390 in colorectal cancer proliferation and metastasis. The results revealed significant (*P* < 0.05) overexpression of lncRNA-XLOC_006390 in colorectal cancer tissues and cell lines, and the transcript levels increased with the advancement of the disease. Moreover, its high expression was shown to be associated with poor patient survival. Silencing of lncRNA-XLOC_006390 in colorectal cancer cells significantly (*P* < 0.05) suppressed their viability via onset of apoptosis and restricted cancer cell migration and invasion. *In vivo* tumor growth was significantly (*P* < 0.05) inhibited under lncRNA-XLOC_006390 repression. LncRNA-XLOC_006390 was shown to sponge the expression of miR-296-3p which in turn acted via post-transcriptional suppression of ONECUT 2 transcription factor to regulate the growth of colorectal cancer. Taken together, the results revealed the oncogenic role of lncRNA-XLOC_006390 in colorectal cancer via modulation of miR-296/ONECUT2 axis. The results also point towards its prognostic and therapeutic potential in the treatment of colorectal cancer.

## 1. Introduction

For their surprisingly immense expression in eukaryotes, the noncoding RNAs were earlier described as the unavoidable transcriptional noise emerging out of exceptionally low reliability of RNA polymerase [1]. However, the advancement in transcriptomics has led to ascription of functional roles to a great majority of noncoding RNAs[2]. The long noncoding RNAs (lncRNAs) constitute a relatively heterogenous class of single-stranded noncoding RNAs arbitrarily greater than 200 nucleotides in size [3]. The lncRNAs are regulatory in function and profoundly affect the expression of specific target genes through varying mechanisms in both the nucleus and the cytoplasm, depending upon their subcellular localization [4]. It is well understood that lncRNAs through their coordination with other molecules affect the eukaryotic physiology and development, and even little functional deviation of lncRNAs has been shown to result in many pathological conditions including cancer [5, 6]. Many lncRNAs regulate one or several key aspects of human tumorigenesis like uncontrolled cell division, avoidance of apoptotic cell death, and metastatic behavior of cancer cells [7]. The cancer biologists have proposed to explore the essence of lncRNAs as biomarkers and feasible therapeutic targets to assist in combating crucial facets of cancer [8]. From the recent research investigations, several lncRNAs were found to be associated with pathogenesis of human colorectal cancer and proved to be involved in either promoting or restricting the growth and metastasis of colorectal cancer cells [9, 10]. Colorectal cancer is highly prevalent in developed countries, annually affecting about 100 million people with mortality rate nearing to 33% [11]. The disease of colorectal cancer is seen with high recurrence and re-appears in half of the patients within five years of disease treatment and results in their death [11]. Scientists are looking at lncRNAs with tremendous possibilities to serve as potential diagnostic and therapeutic biological agents against colorectal cancer [12]. In the present study, the functional role of lncRNA-XLOC_006390 was illustrated in colorectal cancer. LncRNA-XLOC_006390 acts as the crucial regulator of tumorigenesis of human cancers like cervical cancer and pancreatic cancer [13, 14]. However, its functionality is yet to be understood in colorectal cancer. The results of present study were supportive of oncogenic behavior of lncRNA-XLOC_006390 in promoting the growth and metastasis of colorectal cancer cells. LncRNA-XLOC_006390 was shown to be abundantly expressed in colorectal cancer and its silencing induced apoptosis in cancer cells and inhibited their proliferation. The downregulation of lncRNA-XLOC_006390 also restricted cell migration and invasion *in vitro* and restrained the *in vivo* tumor growth. LncRNA-XLOC_006390 was shown to be interacting with and sponging the expression of microRNA-296 (miR-296) in colorectal cancer. The latter was found to be targeting ONECUT 2, a homeobox transcription factor in colorectal cancer. ONECUT 2 acts as a key transcriptional regulator of human tumorigenesis [15]. The present study thus indicated that the enhancement in miR-296 sponging by lncRNA-XLOC_006390, due to overexpression in colorectal cancer, alleviates posttranscriptional targeting of ONECUT 2, and the latter promotes the cancer growth and proliferation.

## 2. Materials and Methods

### 2.1. Collection of Tissue Specimens

The 79 colorectal cancer tissues and adjacent normal tissues were obtained during the period of 2013–2016 from the patients prior to application of radio/chemotherapies at the Second Affiliated Hospital of Harbin Medical University Harbin, China, after informed consent of the patients. The tissues were frozen using liquid N_2_ and stored at ultra-low temperatures for future use. The characteristics of the patients are presented in Table 1. The pathologists of the hospital assisted in the pathological staging of the cancerous specimens. All standard ethical guidelines were followed, and the study was approved by the research ethics committee of the Second Affiliated Hospital of Harbin Medical University Harbin, China, under approval number HMU-3381IV-2020.

### 2.2. Cell Lines and Culture Conditions

A normal epithelial colorectal cell line (CRL-1790) and a panel of colorectal cancer cell lines (CaCo2, HCT-15 and SW-40) were obtained from the Beijing BeiNaChuanglian Biotechnology Research Institute (Beijing, China). Humidified CO_2_ incubator was used for maintaining and propagating the cell lines in RPMI-1640 culture medium (Gibco) supplemented with 10% fetal bovine serum (FBS; Invitrogen; Thermo Fisher Scientific, Inc.) at 37°C.

### 2.3. Transfection

The overexpression vector pcDNA3.1 (Thermo Fisher Scientific) was used for generating the construct ONECUT 2, while empty pcDNA3.1 vector served as expression control. All other expression constructs including si-XLOC_006390, miR-296 mimics, and si-ONECUT 2 together with the respective negative controls were ordered from Gemma Pharmaceutical Technology Co., Ltd. (Shanghai, China).

HCT-15 and CaCo2 cancer cells were seeded in 24 well plates (1 × 10^5^cells/well) in RPMI-1640 media with 10% FBS and antibiotics (penicillin, 100 IU/ml, and streptomycin, 100 *μ*g/ml) at 37°C until they reached 70–80% confluency. Cells were incubated further in serum free media for 1 h and subsequently transfected using Lipofectamine-2000 (Thermo Fisher Scientific) as per the manufacturer's instructions.

### 2.4. Quantitative Real Time-PCR (qRT-PCR)

TRIzol reagent (Thermo Fisher Scientific) was used for extracting total RNA from tissues and cells. For expression analysis of lncRNA-XLOC_006390 and ONECUT 2, cDNA was synthesized with the help of first strand cDNA synthesis kit (Applied Biosystems Inc., Foster City, CA). The qRT-PCR was performed on QuantStudio 3.0 RT-PCR system (Thermo Fisher Scientific) using SYBR Green PCR mix (Thermo Fisher Scientific). The U6 and GAPDH were used as references for the determination of expression. The relative gene expression was calculated by 2^−ΔΔCt^ method. The sequences of the primers used in the study are presented in Table 2.

### 2.5. Cell Viability Assay

The 3-(4,5-dimethylthiazol-2-yl)-2,5-diphenyltetrazolium bromide (MTT) assay was performed for cancer cell viability analysis. Briefly, the transfected cells were cultured with initial cellular density of 10^3^cells per well in 96-well plate. After incubating the plate for 0 h, 24 h, 48 h, and 72 h at 37°C, each well of the plate was added with MTT (2.5 mg/ml) reagent with final concentration of 0.5%, and incubation at 37°C was extended for 4 h again. Following the addition of 200 *μ*l dimethyl sulfoxide (DMSO), the colour developed in samples was read at 570 nm with the help of micro-plate reader (Bio-Rad).

### 2.6. Analysis of Cell Apoptosis

HCT-15 and CaCo_2_ transfected cancer cells were assessed by acridine orange/ethidium bromide (AO/EB) staining method to analyze the levels of cell apoptosis. The cells were cultured in 12-well plate for 48 h at 37°C, fixed with ethanol and then stained with dual staining mix of AO/EB. The AO/EB-stained cells were visualized under fluorescence microscope to detect cell apoptosis. The apoptosis of transfected cells was also determined with the help of Annexin V-FITC/PI apoptosis detection kit as per the manufacturer's guidelines followed by flow cytometric analysis.

### 2.7. Western Blotting

The transfected cancer cells were treated with RIPAlysis buffer (Thermo Fisher Scientific) to isolate total proteins which were quantified by Lowry method. Equal amounts of protein lysates were separated on 10% SDS-PAGE. The PAGE gels were blotted to PVDF membranes, and 5% skimmed milk powder was used to block the membranes. The membranes were subsequently incubated with primary antibodies [Bcl-2 (sc-23960, Santa Cruz, CA, USA), Bax (sc-7480, Santa Cruz, CA, USA), Cleaved caspase-3 (9579, Cell Signalling Technology, Danvers, USA), E-cadherin (3195, Cell Signalling Technology, Danvers, USA), N-cadherin (13116, Cell Signalling Technology, Danvers, USA), MMP-2 (40994, Cell Signalling Technology, Danvers, USA), MMP-9(2270), ONECUT2 (MA5-24275, Thermo Fisher Scientific, Waltham, USA), and *β*-actin (sc-58673, Santa-Cruz, CA, USA)] overnight at 4°C. The membranes were washed three times with TBST and incubated for 2 h with appropriate HRP-conjugated secondary antibody anti-rabbit IgGHRP (7074s, Cell Signaling Technology, Danvers, USA) (Dilution; 1 : 2000). Finally, protein bands were detected through chemiluminescence. Human *β*—actin protein acted as the protein expression control.

### 2.8. Scratch Healing Migration Assay

The migration of transfected colorectal cancer cells was measured with the help of scratch-heal method. The transfected cells were cultured in 6-well plates for 72 h, following which the cell surface was scratched perpendicularly with 10 *μ*l pipette-tip. After 24 h of incubation at 37°C, the width of the scratch was observed and compared with width at 0 h to estimate the cell migration.

### 2.9. Transwell Invasion Assay

The cancer cell invasion was deduced using transwell chambers (Corning, New York, USA). 250 *μ*l of cell suspension in serum-free RPMI-1640 medium carrying 2.5 × 10^5^ cancer cells was added to the upper chamber. The lower chamber was added with 750 *μ*lRPMI-1640 medium supplemented with serum. The transwell chamber plate was incubated at 37°C for 24 h, and then the cells invading the lower chamber were ethanol fixed and subsequently stained with 0.1% crystal violet. Light microscope was used for visualizing the cells, and five random microscopic fields were used for cell counting.

### 2.10. Mice Xenograft Study

The xenograft transplantation of colorectal cancer cells was carried out in 5-week old BALB/c nude mice procured from Chinese Academy of Medical Sciences, Peking Union Medical College, Beijing China. Well aerated rooms were used for maintaining the experimental animals in Animal House. The Animal care and experimentation committee approved the *in vivo* study. The animals were randomly divided into two groups. The CaCo_2_ cancer (5 × 10^6^) cells at exponential growth stage were injected subcutaneous into the dorsal flank of nude mice. The respective mice groups were administered with intratumor injections carrying si-XLOC_006390 or negative control, si-NC. The tumor volume was examined after every 3 days and the mice were finally sacrificed at the end of 3 weeks. The xenograft tumors were then excised; their size and average weight were measured.

### 2.11. Immunohistochemistry (IHC) Assay

The xenograft tumors were excised from each animal, sliced, and fixed in 10% v/v formal saline. The tumors were subsequently embedded in paraffin; sections of 4 *μ*m were prepared. These sections were subjected to IHC staining with anti-Ki67 and anticleaved caspase-3 (Abcam) and mounted on slides previously coated with Mayer's albumin. Cover slip was mounted using DPX and observed using OLYMPUS BX51 microscope, and the images were captured with OLYMPUS DP 72 camera attached to the microscope.

### 2.12. Bio-Informatics Analysis and Dual Luciferase Assay

The predictions of the corresponding molecular targets of lncRNA-XLOC_006390 and miR-296 were made through online bioinformatics. LncBase Experimental v.2 prediction tool was used for predicting the miRNA specifically interacting with lncRNA-XLOC_006390. The mRNA specifically targeted by miR-296 was predicted through TargetScan Human 7.2 (https://www.targetscan.org/vert_72/) online tool. The interaction of lncRNA-XLO_006390 and miR-296 was studied in vitro using dual luciferase reporter assay system (Promega) as per manufacturer's guidelines.

### 2.13. Statistical Analysis

The experiments were performed in triplicate and expressed as mean ± SD. SPSS 22.0 (IBM, SPSS, Chicago, IL, USA) software was used for performing the statistical analyses. Student's *t*-test was used for analyzing the difference between two data sets. *P* < 0.05 was considered as statically significant difference. The Kaplan–Meier method was employed to appraise the survival rate and the analysis was carried out using the log-rank test.

## 3. Results

### 3.1. LncRNA-XLOC_006390 Is Overexpressed in Colorectal Cancer

**Figure 1:**
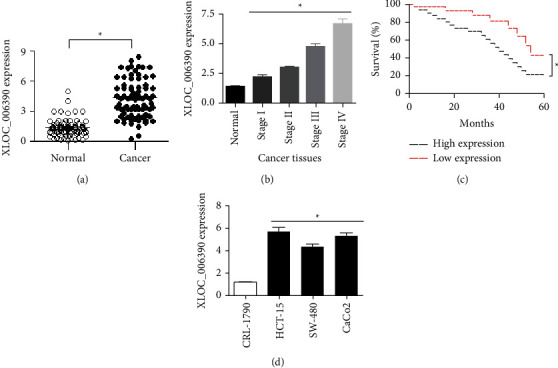
LncRNA-XLOC_006390 is abundantly expressed in colorectal cancer and predicts the patient survival. (a) Dot plot representing the relative transcript level of lncRNA-XLOC_006390 in colorectal cancer tissues in comparison to normal adjacent tissues; (b) relative expression of lncRNA-XLOC_006390 at different pathological stages of colorectal cancer with reference to normal tissue state; (c)Kaplan–Meier analyses of percent survival of colorectal cancer patients with low or high lncRNA-XLOC_006390 expression; (d) relative transcript levels of lncRNA-XLOC_006390 in three colorectal cancer cell lines (CaCo_2_, HCT-15, and SW-40) in comparison to normal colorectal cell line, CRL-1790. The values represent mean ± SD of three biological replicates (^*∗*^*P* < 0.05).

To elucidate the expression of lncRNA-XLOC_006390 in colorectal cancer, qRT-PCR was used. The results showed that the cancer tissues possessed significantly (*P* < 0.05) higher expression of lncRNA-XLOC_006390 in comparison to the normal adjacent tissues (Figure 1(a)). Interestingly, the expression of lncRNA-XLOC_006390 was found to increase with the advancement of colorectal cancer (Figure 1(b)). Based on the expression of lncRNA-XLOC_006390, the patients from whom tissue collection was made were categorized into high expression or low expression group. The survival period was found to be markedly higher for the patients with lower expression of lncRNA-XLOC_006390, while the high-expression patients survived for lesser periods. The relationship between the patient survival and expression of lncRNA-XLOC_006390 was analyzed with the help of Kaplan–Meier test (Figure 1(c)). qRT-PCR was also performed to analyze the expression of lncRNA-XLOC_006390 in colorectal cancer cell lines (CaCo_2_, HCT-15, and SW-40) and normal epithelial colorectal cell line (CRL-1790). All three colorectal cancer cell lines were found to express significantly (*P* < 0.05) higher levels of lncRNA-XLOC_006390 in comparison to the normal colorectal cells (Figure 1(d)). Together, the results suggest that colorectal cancer is associated with lncRNA-XLOC_006390 upregulation, and the expression of lncRNA-XLOC_006390 negatively correlated with the survival of colorectal cancer patients.

### 3.2. Silencing of lncRNA-XLOC_006390 Induced Apoptosis in Colorectal Cancer Cells

**Figure 2:**
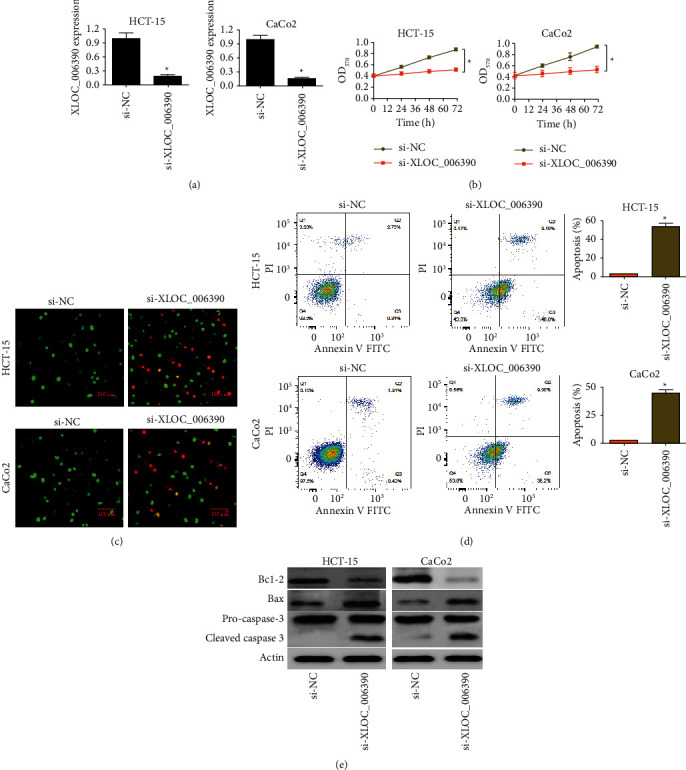
lncRNA-XLOC_006390 downregulation induced cancer cell apoptosis resulting in decline of cell viability. (a) Silencing of lncRNA-XLOC_006390 in HCT-15 and CaCo_2_ cancer cell lines; (b) determination of viability of HCT-15 and CaCo_2_ cancer cells repressing lncRNA-XLOC_006390 and respective control cells by MTT assay; (c) AO/EB staining of HCT-15 and CaCo_2_ cancer cells repressing lncRNA-XLOC_006390 for the estimation of relative cell death with reference to control cancer cells; (d) estimation of apoptosis of HCT-15 and CaCo_2_ cancer cells under lncRNA-XLOC_006390 silencing and respective control cell lines through flow cytometry; (e) western blot analysis of expression of bax, Bcl-2, caspase-3 and cleaved caspase-3 proteins from lncRNA-XLOC_006390 repressing HCT-15, and CaCo_2_ along with respective negative control cells. The values represent mean ± SD of three biological replicates (^*∗*^*P* < 0.05).

To find out whether lncRNA-XLOC_006390 regulates the growth and proliferation of colorectal cancer cells, its expression was silenced in HCT-15 and CaCo_2_ cancer cells, and the same was confirmed by qRT-PCR expression study (Figure 2(a)). The MTT assay showed that silencing of lncRNA-XLOC_006390 suppresses the proliferation of both HCT-15 and CaCo_2_ (Figure 2(b)). The AO/EB staining revealed that lncRNA-XLOC_006390 silencing triggered apoptosis of HCT-15 and CaCo_2_ cancer cells (Figure 2(c)). Similar inference was made by the flow cytometric study where the percentage of apoptotic cells was deduced to be significantly (*P* < 0.05) higher under lncRNA-XLOC_006390 silencing in HCT-15 and CaCo_2_ cancer cells when compared with the negative control cells (Figure 2(d)). The levels of pro-apoptotic proteins like Bax and cleaved caspase-3 were also shown to be enhanced under lnc-XLOC_006390 repression (Figure 2(e)). The results thus support that lncRNA-XLOC_006390 silencing suppresses the proliferation of colorectal cancer via induction of apoptosis.

### 3.3. LncRNA-XLOC_006390 Knockdown Inhibited the Migration and Invasion of Colorectal Cancer Cells

**Figure 3:**
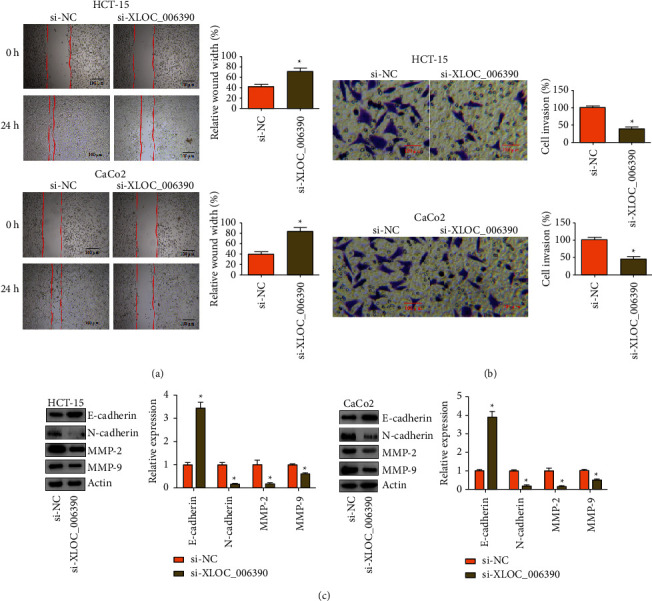
Silencing of lncRNA-XLOC_006390 restricted *in vitro* cancer cell migration and invasion. (a) Analysis of migration of HCT-15 and CaCo_2_ cancer cells repressing lncRNA-XLOC_006390 and control cancer cells by the scratch-heal method; (b) estimation of invasion of HCT-15 and CaCo_2_ cancer cells repressing lncRNA-XLOC_006390 and control cancer cells by transwell chamber assay; (c) western blotting of MMP-2, MMP-9, E-cadherin, and N-cadherin from HCT-15 and CaCo_2_ cancer cells repressing lncRNA-XLOC_006390 and control cancer cells. The values represent mean ± SD of three biological replicates (^*∗*^*P* < 0.05).

The effect of the transcriptional knockdown of lncRNA-XLOC_006390 in colorectal cancer cells was further investigated on the migration and invasion of cancer cells. It was found that silencing of lncRNA-XLOC_006390 caused significant (*P* < 0.05) inhibition of HCT-15 and CaCo_2_ cancer cell migration (Figure 3(a)). The invasion of cancer cells was also reduced significantly (*P* < 0.05) under lncRNA-XLOC_006390 silencing (Figure 3(b)). The western blotting of the metastasis marker proteins revealed that downregulation of lncRNA-XLOC_006390 decreased the expression of N-cadherin, metalloproteinase 2 (MMP-2), and metalloproteinase 9 (MMP-9), while the expression of E-cadherin protein increased (Figure 3(c)). Collectively, lncRNA-XLOC_006390 positively regulates the colorectal cancer metastasis suggesting its therapeutic value.

### 3.4. LncRNA-XLOC_006390 Downregulation Limited in Vivo Growth of Xenograft Mice Tumors

**Figure 4:**
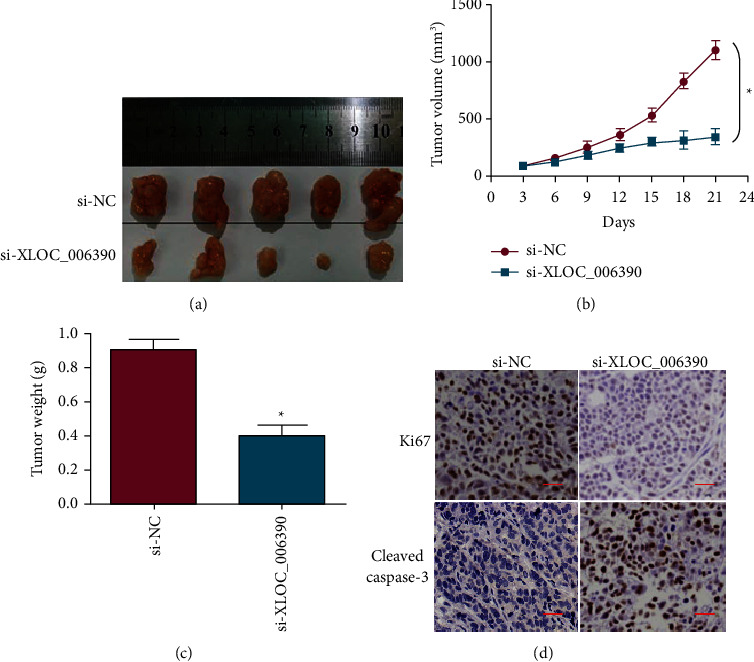
Silencing of lncRNA-XLOC_006390 reduced *in vivo*tumorigenesis. (a) Analysis of relative size of tumor xenografts under lncRNA-XLOC_006390 downregulation with reference to negative control mice tumors; (b) analysis of relative volume of tumor xenografts under lncRNA-XLOC_006390 downregulation with reference to negative control mice tumors; (c) analysis of average weight of tumor xenografts under lncRNA-XLOC_006390 downregulation with reference to negative control mice tumors; (d)immuno-histochemical staining of ki67 and cleaved caspase-3 proteins from tumor xenografts under lncRNA-XLOC_006390 downregulation with reference to negative control mice tumors.

The xenograft mice model was used for assessing *in vivo* role of lncRNA-XLOC_006390 in regulating the tumorigenesis of colorectal cancer. The mice xenografted tumors administered to repress the expression of lncRNA-XLOC_006390 exhibited significantly (*P* < 0.05) lower tumor size in comparison to the negative control mice (Figure 4(a)). The repression was also shown to negatively affect the average tumor volume (Figure 4(b)). After the animal sacrifice, the tumors were excised, and it was found that the average weight was significantly (*P* < 0.05) lower for the mice tumor xenografts downregulating lncRNA-XLOC_006390 as that of the negative control animals (Figure 4(c)). The mice tumors were seen to express higher levels of apoptosis marker protein, cleaved caspase-3, while the proliferation marker protein, ki67, showed exceptionally low expression under lncRNA-XLOC_006390 downregulation (Figure 4(d)). Taken together, the results are supportive of growth regulatory role of lncRNA-XLOC_006390 in colorectal cancer, further indicating it as a potential therapeutic target in colorectal cancer.

### 3.5. LncRNA-XLOC_006390 Sponges miR-296-3p Expression in Colorectal Cancer

**Figure 5:**
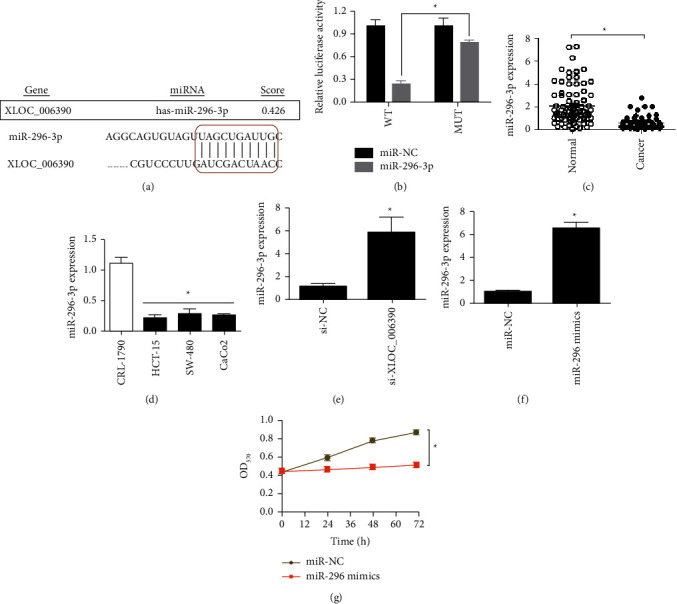
LncRNA-XLOC_006390 sponges miR-296 expression in colorectal cancer. (a)miRNA target analysis of lncRNA-XLOC_006390; (b) dual luciferase assay for the confirmation of target analysis; (c) dot plot of the relative transcript level of miR-296 in colorectal cancer tissues in comparison to normal adjacent tissues; (d) relative transcript levels of miRNA-296 in three colorectal cancer cell lines (CaCo_2_, HCT-15, and SW-40) in comparison to normal colorectal cell line, CRL-1790; (e) expression of miR-296 in CaCo_2_ cancer cells transfected with si-XLOC_006390 or si-NC; (f)over-expression of miR-296 in CaCo_2_ cancer cells; (g) determination of viability of HCT-15 and CaCo_2_ cancer cells over-expressing miR-296 and respective control cells by MTT assay. The values represent mean ± SD of three biological replicates (^*∗*^*P* < 0.05).

The *in silico* analysis predicted that lncRNA-XLOC_006390 interacts in a sequence specific manner with miR-296-3p (Figure 5(a)). To validate the prediction, luciferase reporter assay was performed. The significant (*P* < 0.05) reduction in the luciferase activity of colorectal cancer cells co-transfected with miR-296 mimics and native reporter construct of lncRNA-XLOC_006390, in comparison to that when mutated (MUT) sequence site of lncRNA-XLOC_006390 was used, thereby confirming the interaction betweenlncRNA-XLOC_006390 and miR-296-3p in colorectal cancer (Figure 5(b)). The miR-296-3p expression was found to be significantly (*P* < 0.05) lower in colorectal cancer tissues relative normal tissues (Figure 5(c)). The expression was also significantly (*P* < 0.05) downregulated in the colorectal cancer cell lines in comparison to normal cell line, CRL-790 (Figure 5(d)). Further, the CaCo_2_ cancer cells exhibited significantly (*P* < 0.05) higher transcript levels of miR-296-3p under lncRNA-XLOC_006390 downregulation (Figure 5(e)). The miR-296-3p was overexpressed in CaCo_2_ cancer cells as confirmed by its relative expression (Figure 5(f)). The cancer cells overexpressing miR-296-3p proliferated at minimal rate in comparison to the corresponding control cells (Figure 5(g)). Collectively, the results suggest that lncRNA-XLOC_006390 sponges miR-296-3p expression in colorectal cancer and its overexpression declines miR-296-3p expression to improve the proliferative ability of cancer cells.

### 3.6. LncRNA-XLOC_006390 Regulates ONECUT 2 Expression through miR-296-3p Sponging

**Figure 6:**
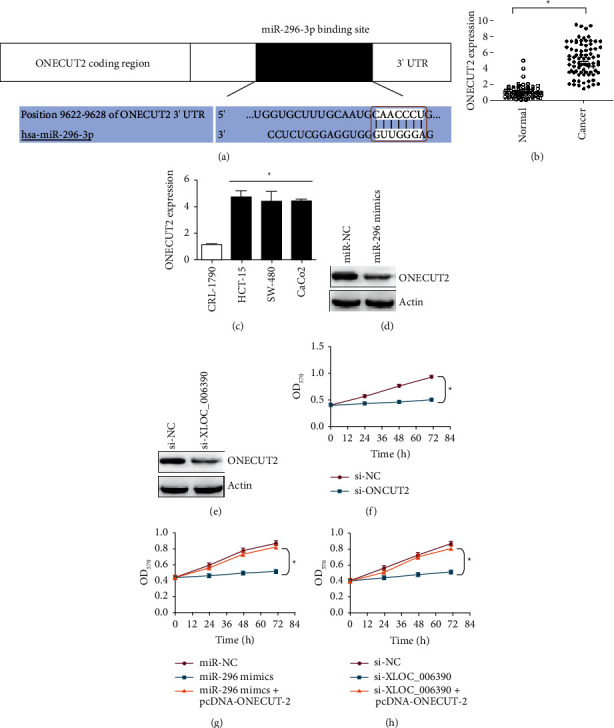
miR-296 targets ONECUT 2 in colorectal cancer. (a) Bio-informatics for the target identification of miR-296; (b) dot plot of the relative transcript level of ONECUT 2 in colorectal cancer tissues in comparison to normal adjacent tissues; (c) relative transcript levels of ONECUT 2 in three colorectal cancer cell lines (CaCo_2_, HCT-15 and SW-40) in comparison to normal colorectal cell line, CRL-1790; (d) western blotting of ONECUT 2 from CaCo_2_ cancer cells transfected with miR-296 mimics or miR-NC; (e) western blotting of ONECUT 2 from CaCo_2_ cancer cells transfected with si-XLOC-006390 or si-NC; (f) estimation of viability of CaCo_2_ cancer cells transfected with si-ONECUT 2 or si-NC by MTT assay; (g) estimation of viability of CaCo_2_ cancer cells transfected with miR-296 mimics, miR-296 mimics plus pcDNA-ONECUT 2 or si-NC b MTT assay; (h) estimation of viability of CaCo_2_ cancer cells transfected with si-XLOC_006390, siXLOC_006390 plus pcDNA-ONECUT 2 or si-NC by MTT assay. The values represent mean ± SD of three biological replicates (^*∗*^*P* < 0.05).

To identify the downstream target of miR-296-3p, online bioinformatics was used. The homeobox transcription factor ONECUT 2 was identified to be the regulatory target of miR-296-3p in colorectal cancer. The miR-296-3p was shown to bind 3′UTR of ONECUT 2 in sequence dependent mode (Figure 6(a)). As expected, the expression of ONECUT 2 gene was significantly (*P* < 0.05) low in cancer tissues and cell lines (Figures6(b) and 6(c)). The levels of ONECUT 2 protein level was found to decrease under miR-296-3p overexpression (Figure 6(d)). Similar reduction in the expression of ONECUT 2 protein was observed under lncRNA-XLOC_006390 silencing (Figure 6(e)). Transcriptional repression of ONECUT 2 in CaCo_2_ colorectal cancer cells declined the cell proliferation significantly (*P* < 0.05) (Figure 6(f)). Conversely, the overexpression of ONECUT 2 in colorectal cancer cells rescued them from the proliferation loss both under miR-296-3p overexpression or lncRNA-XLOC_006390 silencing (Figures 6(g) and 6(h)). Taken together, the results suggest that lncRNA-XLOC_006390 executes its regulatory role in colorectal cancer through miR-296/ONECUT 2 regulatory axis. Both lncRNA-XLOC_006390 and miR-296-3p might be utilized as potential therapeutic targets against colorectal cancer.

## 4. Discussion

Colorectal cancer is ranked as the third most diagnosed human cancer and considered as the fourth most death causing cancer at the global level [16]. The lack of effective prognostic measures and disease recurrence are the dominant factors responsible for higher mortality of colorectal cancer. In depth understanding of various regulatory mechanisms controlling the growth and progression of colorectal cancer is thus indispensable to formulate better screening and treatment procedures against this lethal malignancy. The lncRNA-XLOC_006390 was found to be overexpressed in colorectal cancer in the present study. The overexpression of lncRNA-XLOC_006390 has been shown to be associated with several human cancers, and lncRNA-XLOC_00630 was proven to have oncogenic role in cancer tumorigenesis [14, 17]. Further, it was previously shown that overexpression of lncRNA-XLOC_006390 marks the pathological advancement of human cancer and is linked with the disease metastasis [18]. Supporting the same, the results of our present study showed that the expression of lncRNA-XLOC_006390 increases with the progression of colorectal cancer and promotes the cancer metastasis which highlights the potency of lncRNA-XLOC_006390 to serve in the disease prognosis and therapeutic formulations. LncRNAs are involved in miRNA sponging, and, through this ability, they effectively alter the expression levels of some key miRNA molecules which gets manifested in malignancies like cancer [19]. The sponging of miRNAs by lncRNA-XLOC_006390 has been shown to promote tumorigenicity of cervical cancer [13]. Here, it was revealed that miR-296-3p expression negatively correlated with lncRNA-XLOC_006390 expression, and sponging role of the latter was made clear through *in vitro* experimentation. The miR-296-3p has tumor-suppressive role in colorectal cancer and inhibits the cancer cell metastasis [20]. The lower expression of miR-296-3p was expounded to be involved in chemotherapeutic resistance and marks poor clinical outcomes of colorectal cancer [21]. The miR-296-3p was shown to interact and target ONECUT 2 transcription factor for its translational silencing. ONECUT 2 is a homeodomain type of transcription factor whose expressional elevation promotes the growth and metastasis of colorectal cancer cells [22]. The results of the current study pointed out that colorectal cancer is linked with high ONECUT 2 expressions resulting from miR-296-3p downregulation which in turn emerges from the sponging action of lncRNA-XLOC_006390. In sum, the present work elucidated that overexpression of lncRNA-XLOC_006390 in colorectal cancer leads to repression in the expression of tumor-suppressor miR-296-3p which in turn enhances transcript and protein levels of ONECUT 2 transcription factor, and the latter enables high proliferation and metastasis of colorectal cancer cells.

## 5. Conclusion

Collectively, the current study evaluated the regulatory control exercised by lncRNA-XLOC_006390 on growth and propagation of colorectal cancer. The results showed that lncRNA-XLOC_006390 silencing induces apoptosis of colorectal cancer cells via modulation of miR-296/ONECUT 2 axis. These results point towards the therapeutic implication of lncRNA-XLOC_006390 in the treatment of colorectal cancer.

## Figures and Tables

**Table 1:** Clinical characteristics of colorectal patients that participated in the present study.

Variable	Colorectal cancer patients (*n* = 79)
Age (years)	
** <50**	**31**
** >50**	**48**
Sex	
** **Male	**44**
** **Female	**35**
Differentiation	
** **Well	**17**
** **Moderate	**40**
** **Poor	**22**
Tumor size	
** <5**	**33**
** >5**	**46**
TNM stage	
** **I	**16**
** **II	**18**
** **III	**26**
** **IV	**19**
Lymph node metastasis	
** **Negative	**37**
** **Positive	**42**

**Table 2:** List of primers used in the study.

Primer	Direction	Sequence
XLOC_006390	Forward	5′-CCTTTGAATCCCTGAGAACTGAAC-3′
	Reverse	5′-ACCTTCCTTCCCACTGGACCTTC-3′

miR-296	Forward	5ʹ-TGCCTAATTCAGAGGGTTGG -3ʹ
	Reverse	5′‐CTCCACTCCTGGCACACAG -3ʹ

ONECUT 2	Forward	5′- CATACTCAAGCGGGACCTTCC-3′
	Reverse	5′- TTGGTGGAACTGGGAGTCTAA-3′

U6	Forward	5′- GTCCGGTTTCAGCATGTTT-3
	Reverse	5′-CTCGCTTCGGCAGCACA-3′

GADPH	Forward	5′- CAATGACCCCTTCATTGACC -3′
	Reverse	5′- TGGAAGATGGTGATGGGATT -3′

## Data Availability

The data used to support the findings of the stuyd can be obtained from the corresponding author upon request.
